# The Role of Nutraceuticals and Functional Foods in Skin Cancer: Mechanisms and Therapeutic Potential

**DOI:** 10.3390/foods12132629

**Published:** 2023-07-07

**Authors:** Lucia Peterle, Serena Sanfilippo, Francesco Borgia, Federica Li Pomi, Rossella Vadalà, Rosaria Costa, Nicola Cicero, Sebastiano Gangemi

**Affiliations:** 1 School and Operative Unit of Dermatology, Department of Clinical and Experimental Medicine, University of Messina, Via Consolare Valeria-Gazzi, 98125 Messina, Italy; lucia.peterle@gmail.com (L.P.); fborgia@unime.it (F.B.); federicalipomi@hotmail.it (F.L.P.); 2 School and Operative Unit of Allergy and Clinical Immunology, Department of Clinical and Experimental Medicine, University of Messina, Via Consolare Valeria-Gazzi, 98125 Messina, Italysebastiano.gangemi@unime.it (S.G.); 3 Department of Biomedical and Dental Sciences and Morphofunctional Imaging, University of Messina, 98168 Messina, Italy; rossella.vadala@unime.it (R.V.); costar@unime.it (R.C.); 4 Science4life srl, University of Messina, 98168 Messina, Italy

**Keywords:** skin cancer, nutraceuticals, omega-3 fatty acids, polyphenols, isoflavones, melanoma, non-melanoma skin cancer, chemoprevention, skin cancer treatment

## Abstract

Skin cancer is a prevalent type of cancer worldwide and has a high growth rate compared to other diseases. Although modern targeted therapies have improved the management of cutaneous neoplasms, there is an urgent requirement for a safer, more affordable, and effective chemoprevention and treatment strategy for skin cancer. Nutraceuticals, which are natural substances derived from food, have emerged as a potential alternative or adjunctive treatment option. In this review, we explore the current evidence on the use of omega-3 fatty acids and polyphenols (curcumin, epigallocatechin gallate, apigenin, resveratrol, and genistein) for the treatment of melanoma and non-melanoma skin cancer (NMSC), as well as in their prevention. We discuss the mechanisms of action of the aforementioned nutraceuticals and their probable therapeutic benefits in skin cancer. Omega-3 fatty acids, curcumin, epigallocatechin gallate, apigenin, resveratrol, and genistein have several properties, among which are anti-inflammatory and anti-tumor, which can help to prevent and treat skin cancer. However, their effectiveness is limited due to poor bioavailability. Nanoparticles and other delivery systems can improve their absorption and targeting. More research is needed to evaluate their safety and effectiveness as a natural approach to skin cancer prevention and treatment. These compounds should not replace conventional cancer treatments, but may be used as complementary therapy under the guidance of a healthcare professional.

## 1. Introduction

The relationship between diet and tumor development is complex and multifaceted. Epidemiological and clinical studies have shown that dietary mutagens can lead to alterations in normal biological pathways, which can contribute to the development of neoplasms. On the contrary, certain dietary components have been found to protect against cancer by promoting DNA metabolism and repair and preventing damage due to oxidative stress [1].

Skin cancer is the most prevalent neoplasm worldwide and has a high growth rate compared to other diseases [2]. Skin cancer includes two main classes: cutaneous melanoma and non-melanoma skin cancer (NMSC), namely basal cell carcinoma (BCC) and squamous cell carcinoma (SCC). NMSC originates from keratinocytes, while melanoma arises from melanocytes. Several mechanisms contribute to the pathogenesis of cutaneous tumors and in their progression [3,4,5]. Among these, oxidative stress (OS) plays a pivotal role [6], as it does in other skin diseases [7,8], and is often exploited for therapeutic purposes [9,10]. Melanoma has the worst prognosis and a high mortality for skin cancer. Melanoma cells have been shown to have adaptive mechanisms that allow them to exceed the effects of high levels of reactive oxygen species (ROS), which are produced in cells during ordinary metabolic processes or in response to environmental stressors [6].

Melanoma progression requires multistep changes in melanocyte characteristics, including the acquisition of the ability to proliferate, survive, and metastasize [2]. The current treatments for skin cancer are very expensive, quite toxic, and sometimes ineffective due to resistance. Therefore, it is required to figure out new, affordable, and effective therapeutic strategies, such as medicinal plants that have a thousand-year history as therapeutic tools for many diseases [11]. The term “nutraceutical”, formed by combining the words “nutrition” and “pharmaceuticals”, is known as any natural compound derived from food or part of it that provides a medical advantage [12]. Studies have shown that some of these natural extracts may exert anti-inflammatory, antioxidant, and antiproliferative effects [13], demonstrating their possible use for the treatment of different cutaneous diseases [14,15].

Various bioactive food compounds, including polyphenolic compounds, such as curcumin, resveratrol, apigenin, rosmarinic acid, and anthocyanins [16,17,18,19,20,21], etc., fatty acids, [22] and micronutrients, have been found to have a protective function against tumor development and they are useful as an adjuvant treatment.

Chemoprevention involves the employment of natural or synthetic chemical compounds that can slow down or reverse carcinogenesis. Chemoprevention is a pivotal approach to cancer control, offering a tool for decreasing the risk of cancer development. It is notably relevant in the context of skin cancer, where exposure to multiple predisposing environmental factors can be difficult to control [23].

The aim of this review is to assess several current nutraceuticals, in particular omega-3 fatty acids and polyphenols, in order to:

(1) clarify their mechanism of action;
(2) and evaluate their possible use in the treatment and prevention of melanoma and NMSC.

## 2. Discussion

### 2.1. Fatty Acids and Skin Cancer

Fatty acids (FA) are components of the cell membrane and key actors in the transduction signals in the body. There are several types of FAs with multiple physiological and pathological functions. FAs are categorized into three main classes based on their varying degrees of saturation: saturated fatty acids (SFA), monounsaturated fatty acids (MUFA), and polyunsaturated fatty acids (PUFA) [24]. PUFAs are further classified into ω-3 and ω-6 groups, according to the location of the first double bond from the methyl terminal of the FA. These structural diversities lead to functional distinctions in terms of their effects on inflammation and metabolism [25]. ω-3 PUFAs are essential nutrients, making it mandatory to consume foods rich in ω-3 (such as cold-water fish, nuts, and seed oils) [26]. Animal and in vitro studies have suggested that n-3 fatty acids, especially eicosapentaenoic acid (EPA) and docosahexaenoic acid (DHA), may have anti-cancer properties. One proposed mechanism is that these FAs can reduce inflammation in the body. In addition, EPA and DHA have been demonstrated to inhibit the growth and proliferation of malignant cells in laboratory studies [27,28].

Regarding the skin, Storey et al. demonstrated that ω-3 PUFAs may have anti-inflammatory action in skin cells exposed to UVB radiation, one of the principal risk drivers for melanoma and NMSC. Specifically, the researchers found that ω-3 PUFAs may be able to reduce interleukin-8 (IL-8) expression by modulating tumor necrosis factor- α (TNF-α), another pro-inflammatory cytokine. Furthermore, the researchers compared the relative potencies of two key ω-3 PUFAs, eicosapentaenoic acid (EPA) and docosahexaenoic acid (DHA). The results suggested that EPA and DHA may be effective in modulating IL-8 expression and reducing inflammation in skin cells, although EPA appeared to be somewhat more potent than DHA in this regard [29].

In addition, although no evidence has been found to suggest that EPA directly protects against the formation of cyclobutane pyrimidine dimers, a type of DNA injury caused by ultraviolet radiation (UVR) exposure, EPA does provide protection against the induction of cutaneous p53, which is a tumor suppressor gene and a biomarker of DNA damage. Moreover, ω-3 PUFAs, including EPA, were found to protect against DNA single-strand breaks in peripheral blood lymphocytes that had been exposed to UVR ex vivo [30]. Rhodes et al. wanted to evaluate the dietary photoprotection provided by EPA in a double-blind randomized trial. The study demonstrated that EPA provides a considerable protection effect against UVR-induced erythema in humans. EPA guards against the visible signs of sunburn, but also against UVR-induced p53 expression [30]. Later, the same group, a randomized controlled study was conducted with 79 volunteers to examine the impact on the UVR suppression of cutaneous cell-mediated immunity (CMI) reflected by nickel contact hypersensitivity (CHS). They demonstrated that oral ω-3 PUFAs seem to repeal photoimmunosuppression in human skin, supporting their chemopreventive function [31]. In addition, they wanted to clarify if EPA reduces skin inflammation via competition with arachidonic acid (AA) for metabolism by cyclooxygenases/lipoxygenases. They examined the effect of EPA intake on AA level, EPA, and their resulting eicosanoids in human skin with or without UVR. They proved that dietary supplementation with EPA increased the levels of EPA in erythrocytes and the skin while lowering the ratio of AA to EPA. Before supplementation, exposure to UVR increased the generation of pro-inflammatory eicosanoids, such as PGE2, 12-HEPE, and PGE3. However, after the EPA supplementation, there was a reduction in PGE2 in unchallenged skin and an increase in EPA-derived PGE3 and 12-HEPE after UVR exposure. This shift towards less pro-inflammatory eicosanoids was observed in both basal conditions and subsequent to an inflammatory insult, suggesting that EPA promotes a regulatory environment in the skin [32]. In another study, they examined if EPA ingestion modifies the UV-mediated effects on epidermal Langerhans cell (LC) numbers and the levels of PG D2, which is produced by LCs. This study did not support the influence of EPA on immunoprotective effects through changes in the number of epidermal LCs, although it is probable that there were changes in the LC activity [33].

Serini et al., in 2016 demonstrated that DHA suppresses melanoma cell growth, migration, and invasion, respectively, increasing nuclear b-catenin content, nuclear expression, PKA-dependent phosphorylation, and microphtalmia transcription factor (MITF) [34].

The protective effect of ω-3 in preventing the development of skin tumors could therefore be utilized for prevention in patients who have risk factors for tumor development, such as transplant recipients. In this sense, Miura K. et al. demonstrated a decrease in new skin cancer cases in transplant patients taking ω-3 supplementation, supporting the idea that a more extensive and conclusive trial is needed [35].

In support of this, Donat-Vargas, in 2015, conducted a prospective study on 20,785 women, evaluating with a questionnaire their EPA-DHA intake and evaluating the incident melanoma cases. In women with the highest tertile of EPA-DHA intake, an 80% lower risk of melanoma was observed than in those with the lowest tertile, showing a statistically significant difference [36]. If a possible association has been found for melanoma, it has not yet been established for BCC and SCC. In fact, in a study conducted by Wallingford et al. in 2012, aimed at investigating a possible association between the consumption of ω-3 and ω-6 and the risk of BCC and SCC, no association was found [37].

While fatty fish and fish oils have been shown to have possible health advantages, it is important to weigh these benefits against the potential risks of consuming high levels of environmental pollutants, such as mercury and polychlorinated biphenyls (PCBs), which may be found in these foods. To reduce exposure to these contaminants, it is recommended to consume fish in moderation and choose lower-mercury varieties (such as salmon, sardines, and trout). Alternatively, supplements can be taken to obtain ω-3 [38]. The recent finding that higher fish consumption is associated with an augmented risk of squamous cell carcinoma in Singapore is concerning. It is plausible that this association could be correlated with higher levels of arsenic in seafood, since arsenic is a known carcinogen and can be found in certain types of fish and shellfish [39].

### 2.2. Dietary Polyphenols and Skin Cancer

Polyphenols are natural compounds found in several plant-based foods such as berries, tea, coffee, dark chocolate, and vegetables. They have powerful antioxidant and anti-inflammatory properties that can support the immune system and potentially reduce the risk of cancer and aging. Well-studied polyphenols, including curcumin, epigallocatechin-gallate, apigenin, resveratrol, and genistein, have shown potential in preventing and treating cancer by reducing inflammation and oxidative stress [40].

#### 2.2.1. Curcumin and Skin Cancer

Curcumin is polyphenolic extracted from the rhizome of *Curcuma longa* and it is a yellow bioactive compound, the consumption of which has increased rapidly over the last few years due to its important effects (antioxidant, anti-inflammatory, antimicrobial, antitumor, neuro-, and hepatoprotective).

In recent years, the antitumor role of curcumin in melanoma has been evaluated through preclinical studies on animal models. Several molecular mechanisms are involved: cell cycle alteration (cyclins A, B, D1, and E), apoptosis (caspases-3, -8, -9, Bax, and mTOR vs. Bcl-2, Bcl-XL, and XIAP), interaction with growth factors (TNF-α, IFN-γ, and IFN-α), kinases (IKK, JAK, Akt, PI3K, ERK, MAPK, and CHEK2), and transcription factors (NF-kB, STAT-3, p53, p21, p65, and AP-1), and chemotactic, angiogenic, and metastatic factors (VEGF, COX, MMP, LOX, and NOS). However, the design of clinical trials is impaired by the low oral bioavailability and chemical instability of curcumin, so more readily available derivatives are being created [41,42].

The antiproliferative and pro-apoptotic role of curcumin has been demonstrated in several in vitro studies, showing no limitation of toxicity in phase I and II. In animal studies, curcumin has caused tumor inhibition and the induction of apoptosis in numerous types of malignancies (skin, colon, liver, ovary, breast, gastric, and pancreas) [43,44].

The known cytotoxic action of curcumin is enhanced by the addition of tocopherol (tocopherol polyethylene glycol succinate o TPGS) on nanopattern films, both in vitro and in vivo, in SCC [45]. Jose et al., in 2017, studied the association between curcumin and STAT3-siRNA loaded on cationic liposomes. They noted that free curcumin did not cause a significant decrease in STAT3 protein expression, while curcumin loaded on liposomes showed a reduction in STAT3; this suppression was amplified using co-loaded curcumin and STAT3-siRNA liposomes [46]. In 2018, the same group of researchers noted that a combination of curcumin and STAT3-siRNA complexed in liposomes would appear to enhance the tumor growth suppression in melanoma through the inhibition of STAT3. The latter is a transcription factor implicated in melanoma progression, as it promotes the expressions of VEGF, BCL2, IL-17, and IL-23 [46]. In a cell model of SCC, STAT3 inhibition reduces the growth and invasive ability of cancer cells using curcumin at different doses [47]. The use of curcumin-silica nanoparticles as photosensitizers for photodynamic treatment enhances the photocytotoxicity against melanoma, probably due to an increase in ROS production. In addition, the cytotoxicity on fibroblasts and no structural change in hemoglobin were excluded [48]. Phillips et al., in 2013, demonstrated the potential chemopreventive role of curcumin in skin carcinogenesis; they observed that topical or oral administration of curcumin inhibited the multiplicity formation of tumor lesions in daily UVB-radiated mice and delayed the appearance of potential skin tumors [49]. Another study about the potential chemopreventive role of curcumin is the study conducted by Tremmel et al., in which a combination of ursolic acid and curcumin was applied topically and determined an inhibition of epidermal EGFR, p50, NF-κB, p70S6K, Src, Rb, c-Jun, and IκBα and the levels of c-Fos, Cox-2, and c-Junwere also significantly reduced [50]. In 2018, Singh et al. demonstrated the efficacy of curcumin-loaded liposome gold nanoparticles as in situ adjuvants in photothermal therapy for melanoma, compared with free curcumin and curcumin-loaded liposomes [51]. In agreement with this result, in 2020, nanoparticles of selenium-polyethylene glycol-curcumin (Se-PEG-Cur) were created and have been shown to be an effective absorbing agent for both the photothermal therapy (PTT) and sonodynamic therapy (SDT) of melanoma by increasing ROS production [52]. Demethoxycurcumin (DMC) is a derivative of curcumin, with the same biological properties but a greater chemical stability. The use of DMC determines cell cycle arrest in the G2/M phase and inhibits the viability of A431 and HaCaT cells, resulting in a reduction in Bcl-2 levels and an augmentation of BAX, caspase-9, caspase-3, and cytochrome c. It has been hypothesized that DMC regulates cytochrome c release, mediated by Bcl-2 and BAX, which activate caspases-9 and -3, promoting apoptosis [53]. In a study by Jiang et al., curcumin appears to have suppressed NF-kB and the related genes that are responsible for tumor cell proliferation and apoptosis (such as Mcl-1 and Bcl-2). It also appears to have increased the expression of Bax, a target of p53. Bax and Bcl-2 are pivotal actors for apoptosis or cell survival. In addition, they found a downregulation of p38, indicating a potential role of the p38-MAPK pathway in curcumin-mediated apoptosis. Thus, the regulation of the expressions of Bax, Bcl-2, and Mcl-1 demonstrates the role of the mitochondrial pathway as the main mechanism of curcumin-induced apoptosis, while the cleavage of caspase-3 and caspase-8 also suggests an activation of the extrinsic pathway [54]. Comparable results were found in an in vitro study conducted by Niu et al., in which low doses of curcumin and red united blue light irradiation were used to treat melanoma [55] and in another study by Zhao et al., in vitro and on mice in melanoma models; both studies observed an increase in LC3, suggesting increased autophagic activity [56]. It appears that the antiproliferative effect of curcumin is mediated by ROS production, while the apoptotic effect is due to the induction of the mitochondrial pathway [57]. Khandelwal et al. demonstrated that the use of curcumin C3 complex associated with an FGFR inhibitor (AZD4547) reduces the cell proliferation and progression in the cell cycle, inducing a reduction in the hyperplasia and hypertrophy in JB6 cells and UVB-irradiated SKH-1 mice. FGF2 binds phosphorylated FGFR2, leading to STAT, Ras-dependent, and Ras-independent MAPK and PI3-AKT pathways, but an increase in mTORC1 and mTORC2 was also observed; this suggests an interaction between the FGFR and mTOR signaling pathways. In agreement with the activation of the PI3K/AKT/mTOR/P70S6K pathway is also the study by Zhao et al. [56,58]. Curcumin’s bioavailability and biodisponibility is limited due to several factors [59]; for this reason, Faião-Flores F. and coworkers, in 2013, studied the action of a curcumin analogue (DM-1) in an in vitro model of melanoma, observing that its antitumor action would appear to be due to a loss of the normal cytoskeleton structure by the retraction of filopodium, structural alterations in some proteins (anoikis), and the induction of Bcl-2 proteins that will activate apoptosis via intrinsic and extrinsic pathways [59]. In 2015, the same group conducted a study on mice with melanoma, administering DM-1 as a single dose or combined with dacarbazine (DTIC); they observed a reduction in tumor mass with increased survival and effects on cancer-induced anemia (normalization of RBC, WBC, and platelets [59].

In 2017, Kim et al. treated a 43-year-old female patient with mycosis fungoides, which had been resistant to previous conventional treatments, with a curcuminoid essential oil complex (CEC). Within two weeks, they observed a reduction in the tumor lesion, with reductions in erythema and induration; the itching was reduced and the small lesion near the main disappeared fully [60].

#### 2.2.2. Epigallocatechin Gallate and Skin Cancer

Catechins are polyphenolic derivatives belonging to the flavonoid family (a subclass of polyphenols), which are found in a variety of plants. Green tea, cocoa-based products, and wine are the major sources of these flavonoids. Catechin, epicatechin (EC), epicatechin gallate (ECG), epigallocatechin (EGC) and its stereoisomer gallocatechin (GC), and epigallocatechin gallate (EGCG) and its stereoisomer gallocatechin gallate (GCG) are the main constituents of catechins, and their compositions exhibit a high degree of similarity [61].

Green tea is full of catechins, and epigallocatechin-3-gallate (EGCG) is the most biologically active compound [62]. The high biological activity of EGCG is due to a trihydroxyl structure in the D ring (gallate) [63]. EGCG possesses anti-inflammatory, antioxidant, and antitumor properties in several organs [64]. Many studies have proved the potential action of EGCG, both in the prevention of and as a possible treatment for skin tumors. Regarding prevention, Chiou et al. demonstrated that peracetylated (Ac)-EGCG was a CD34(+) and PKD1 inhibitor in a mouse skin carcinogenesis model, suggesting that Ac-EGCG has the potential to be engineered into a novel chemopreventive agent, and that PKD1 could be a target for both the prevention and treatment of skin cancer in clinical settings [65]. In addition, Sarkar et al. demonstrated that EGCG was able to increase the viability of human keratinocyte cells treated with arsenite (AsIII), a compound known for prompting OS and skin carcinogenesis by partially restoring the Nrf2/Keap1-mediated signaling axis involved in the cytoprotective responses to exogenous and endogenous OS [66]. Balasubramanian et al., given that Bmi-1 is an important member of the polycomb group PcG family that is overexpressed in skin tumor cells and a target of EGCG, wanted to understand the role of its functional domains in the survival of malignant cells. They discovered that the RF and HT domains of Bmi-1 are necessary to counteracting the anti-tumor effects of EGCG, providing a deeper understanding of the molecular pathways underlying the anti-tumor effects of EGCG, the role of Bmi-1 in promoting cell survival, and new avenue for developing drugs that target these specific domains and inhibit the Bmi-1 activity in skin tumor cells [67].

In addition to this, Singh et al. found that β-catenin signaling is a possible target of EGCG, since the treatment of the A431 and SCC13 skin cancer cell lines with EGCG showed a significant suppression of cell viability and a subsequent marked inactivation of β-catenin [68].

Regarding melanoma, EGCG significantly reduces the interaction between TNF Receptor-Associated Factor 6 (TRAF6) and UBC13(E2) and suppresses TRAF6 E3 ubiquitin ligase activity, both in vivo and in vitro. Furthermore, EGCG treatment results have shown a decrease in the phosphorylation of IκBα, p-TAK1 expression, and the nuclear translocation of p65 and p50, causing the inhibition of the NF-κB pathway. In addition, EGCG significantly inhibits the cell growth, invasion, and migration of melanoma [69].

In another study conducted on the mouse melanoma cell line B16F10, Xu et al. demonstrated that a combination of EGCG and metformin had a synergistic role in cell viability, proliferation, and migration, as well as in the signaling of the transcription 3/nuclear factor-κB (STAT3/NF-κB) pathway, and, consequently, in inflammation cytokine production. These results provided new insights into the potential anti-tumor effects of EGCG on melanoma cells, particularly in combination with metformin [70]. In another study by Roomi et al., EGCG downregulated, in a dose-dependent manner, the expression of the matrix metalloproteinase (MMP)-2 and -9 secretions that destroy the extracellular matrix and basal membrane, allowing malignant cells to spread to distal organs [71]. El-K.ayal et al. found that EGCG loaded into ultradeformable colloidal vesicular systems showed an inhibitory action on an epidermoid carcinoma cell line (A431), reducing the tumor sizes in mice, which was confirmed with a histopathological analysis and biochemical biomarkers (glutathione, catalase, superoxide dismutase, and lipid peroxidation) [72]. Liao et al. developed EGCG-nano ethosomes for transdermal delivery and assessed them for treating subcutaneously implanted human melanoma cells in mice. The mice treated exhibited a significant therapeutic effect, demonstrated by their tumors shrinking [73].

Despite primarily functioning as an antioxidant, EGCG has demonstrated a pro-oxidant effect due to its molecular structure [74]. Based on this, León et al. demonstrated in a squamous cell carcinoma model (HSC-1 cells) resistant to MAL-PDT that EGCG is a PDT enhancer in this model.

#### 2.2.3. Apigenin and Skin Cancer

Apigenin is a natural substance belonging to the subclass of flavonoids that is found in various plants belonging to the Artemisia family (such as Tanacetum, Achillea, Artemisia, and Matricaria) [75].

Apigenin has been found to have anti-tumor activity and has shown an effectiveness in suppressing cell growth in various types of human cancer cell lines, including leukemia, prostate, thyroid, colon, and breast cancer cells [76,77,78].

Apigenin’s anticancer effects may be due to its capacity to induce cell cycle arrest, inhibit angiogenesis, induce apoptosis, and suppress the activity of the various signaling pathways that regulate cancer cell growth and metastasis [18]. Regarding the skin, studies have shown that apigenin has a possible role in the tumorigenic response of skin cancers. Tong et al., in 2014, for the first time, identified the mechanisms by which apigenin inhibits UVB-induced skin carcinogenesis in in vitro and murine models. They demonstrated that apigenin increases the de novo synthesis of an angiogenesis inhibitor, thrombospondin-1 (TSP1), in keratinocytes exposed to UVB, and it normalizes proliferation and angiogenesis in UVB-exposed skin [79]. Later, the same research group demonstrated that TSP1 acts a critical role in apigenin-mediated chemioprevention in UVB-induced skin cancer. Notably, in mice lacking TSP1 (TKO mice), there was increased baseline inflammation in the skin, characterized by higher levels of inflammatory cells (neutrophils and macrophages) and two important cytokines involved in inflammation, IL-6 and IL-12. Conversely, applying apigenin topically to the skin of normal mice (WT mice) with maintained TSP1 expression after UVB irradiation led to a decrease in circulating inflammatory cytokines, suggesting that the anti-tumor role of apigenin in the skin is due to its anti-inflammatory action [80]. Another study provided by Kiraly et al. showed that apigenin reduced the synthesis of COX-2, PGE2, EP1, and EP2, while also increasing the terminal differentiation in normal skin cells. However, in tumoral cells, apigenin did not inhibit the COX-2 pathway or promote terminal differentiation. Based on these results, the authors suggested that apigenin may prevent skin tumor growth by blocking COX-2 [81]. Kubik et al. found that Centaurea castriferrei Borbás & Waisb is rich in apigenin and its derivatives. They demonstrated that extracts from these plants have a cytotoxic effect against all tested cell lines, including melanoma cells, without causing a significant decrease in the viability of normal fibroblast cell lines [82]. Another study provided by Bridgeman et al. demonstrated that another target of apigenin is the mTOR signaling pathway, which is hyperactivated in NMSC [83]. In another study provided by Li et al., apigenin was able to restore the inhibition of autophagy in UVB-exposed human epidermal keratinocytes (HEKs). Additionally, apigenin restored the downregulation of many regulatory proteins, such as ATM, ATR, IRE1α, BiP, and PERK, in HEKs exposed to UVB radiation. Apigenin treatment also inhibited UVB-induced apoptosis and cell death in HEKs, suggesting its possible application as a photoprotective agent [84].

In addition, García-García et al. demonstrated that apigenin inhibits IKKα expression, a protein that promotes tumor growth and metastasis by inducing epithelial–mesenchymal transition (EMT) in murine models of SCC, suggesting a possible role of apigenin in the treatment of these cancers [85]. Another target of apigenin in SCC is Sulfiredoxin (Srx), a protein with an oncogenic role in skin tumorigenesis. A study by Wang et al. showed that apigenin induces apoptosis by downregulating Srx through the MAPK pathway in SCC cells [86]. Regarding the regulation of oxidative stress, Paredes-Gonzalez et al. found that apigenin can reverse the hypermethylation of 15 CpG sites in the Nrf2 promoter in skin epidermal JB6 P+ cells. This hypermethylation of the promoter region has previously been shown to silence the Nrf2 gene, which is a pivotal regulator of antioxidant and detoxifying enzymes [87].

Given the evidence for a possible therapeutic role of apigenin in skin tumors, many studies have focused on the possible use of nanoparticles as carriers for this molecule. In a study provided by Waheed et al., lyotropic liquid crystalline nanoparticles (LLC NPs) containing apigenin (API) were developed using a quality by design (QbD) approach to enable efficient permeation and enhanced bioavailability for dermal delivery. The dose-dependent effectiveness of these API-LLC NPs was demonstrated in melanoma cell lines (B16F10), indicating the potential of these developed nanoparticles to target the deeper skin layers [88,89]. Das S. and his research group loaded apigenin in poly (lactic-co-glycolide) (PLGA) nanoparticles (NAPs) to determine if nano-encapsulation could improve the anti-carcinogenic effect against skin tumors induced by ultraviolet B (UVB) and Benzo(a)pyrene (BaP) and mitigate mitochondrial dysfunction in mice. NAp was shown to decrease tissue injury and chromosomal aberrations, increase ROS, and modulate many apoptotic markers and mitochondrial matrix swelling, ultimately resulting in mitochondrial-mediated apoptosis [89]. In addition, the same research group evaluated the effects of NAp on the stability of the dsDNA of melanoma cells (A375). The results showed that NAp could easily enter cancer cells, interact with dsDNA, and induce conformational changes, leading to increased levels of ROS and a reduction in antioxidant enzymes, contributing to DNA injury and apoptosis through mitochondrial dysfunction, also in melanoma cell lines [90]. Jangdey M.S. et al. formulated a nanoemulsion gel based on Arbopol and containing apigenin using tamarind gum emulsifier, which resulted in a small droplet size, high drug content, and good physical stability for efficient release to the skin. The gel demonstrated toxicity in melanoma cells (A341) and less toxicity toward HaCaT cells [91]. The same research group optimized transferosomes using a Box–Behnken design, obtaining a formulation with a good stability, sustained release of apigenin over a prolonged period [92], and a Carbopol-based nanotransfersomal gel loaded with apigenin. This conjugated formulation showed good size, zeta potential, polydispersity index, and % conjugation efficiency, exhibiting toxicity in melanoma (A375) cells, but less toxicity towards HaCaT cells [93].

#### 2.2.4. Resveratrol and Skin Cancer

Resveratrol is a non-flavonoid polyphenol belonging to stilbene phytoalexin, found mainly in red grapes, berries, and peanuts; it can exist in two forms (cis- and trans-). It is a compound produced as a defense mechanism by various plants in the cases of parasitic infections, fungal infections, UV radiation, chemicals, and stressors [94].

The role of resveratrol has been studied for several years for its various properties in many diseases (cancer, cardiovascular diseases, viral infections, and neurodegenerative diseases), showing potent anti-inflammatory, antioxidant, and antiproliferative actions [95].

Several studies have identified the main skin antitumor mechanisms of resveratrol: oxidative DNA damage (LPO), cell cycle regulation (cyclins, cyclin kinase inhibitors, and cyclin-dependent kinases), apoptosis (survivin, p53, Smac/DIABLO, and M30-CycDEATH), tumor initiation and invasion, metastasis (PCNA, NF-kB, and Ki-67; COX-2 and ODC; E-Cadherin, Akt, TGF-β2, and pCREB), and autophagy (light chain 3 and Rictor) [96,97].

Many studies have shown the chemopreventive action of resveratrol in the process of the carcinogenesis of different organs (skin, breast, pancreas, lung, prostate, and ovary) [98,99,100].

Resveratrol has been shown to inhibit and reverse the process of the epithelial–mesenchymal transition (EMT-)-related morphologic modifications caused by the TGF-β, NF-κB, TGF-β/Smads, PI3K/Akt, EGF, and Hedgehog pathways and it also has a chemopreventive role in the invasion and migration of tumors by suppressing the related pathways such as MMPs, VCAM-1, HGF, andHER-2/neu [101].

Unfortunately, resveratrol is poorly bioavailable, so its intestinal absorption is low; hence, specific formulations have been created to promote greater absorption [102].

Topical resveratrol and quercetin associations loaded on nanostructured lipid carrier (NLC) gel or in liposome have been used in some studies, which have shown improved bioavailability and efficacy, especially in those skin cancers resistant to conventional therapy [103,104,105]. This technique has also been used for other associations (such as resveratrol and ω-3) [105].

The design of copolymer nanoparticles to deliver resveratrol allows a better therapeutic bioavailability of resveratrol and shows greater antioxidant action than free resveratrol [106].

The action of solid lipid nanoparticles loaded with curcumin and resveratrol on melanoma cells has been studied and a reduction in tumor cell migration and viability was noticed [54,107].

A blend of resveratrol and 5-fluorouracil loaded onto ultradeformable liposomes was studied by Cosco et al. in cells with nonmelanoma skin cancer; a G1/S cell cycle stop and a reciprocal enhancement of the antitumor action of the two compounds was observed [108].

Zhang et al. observed that the use of resveratrol as an adjuvant for PDT potentiates the effect of tumor growth inhibition in cells with SCC; the pathway involved would appear to be that of p38/MAPK [86].

Wu et al. designed transfersomes to deliver resveratrol and make it more stable and penetrating on the skin. They showed no improvement in antioxidant activity after the encapsulation in transfersomes compared to free resveratrol; however, they observed a better skin penetrability and a smaller cytotoxic effect in the transfersomes loaded with resveratrol [94]. In a study on melanoma cells, the use of resveratrol in combination with chloroquine resulted in decreased beclin-1 and thus autophagy and in increased, unused LC3II; these results seem to be mediated by the inhibition of NF-kB and p62 by resveratrol [109].

In a recent study, resveratrol and two of its derivatives (ε-viniferin and labruscol) were tested in vitro on melanoma cells; first, a cytostatic effect was observed, followed by a cytotoxic effect, with subsequent cell death via apoptosis and necrosis on tumor cells and not on fibroblasts. In this study, it was observed that resveratrol reduces cyclin D1 and increases cyclin A2, E1, Cdk1, and Cdk2, blocking the cell in S phase. The two resveratrol derivatives showed similar but less pronounced activity [110,111]. Fang et al. evaluated the role of resveratrol as a radiation sensitizer against melanoma cells; they observed a suppression of cell proliferation (reduced expressions of cyclin B and D and Cdk2 and 4) and the promotion of apoptosis (reduced expressions of FLIP, Bcl-2, and survivin), as in their earlier study on radioresistant prostate cancer [111].

A recent work on melanoma cells demonstrated a significant increase in apoptosis after resveratrol use. This may be due to the downregulation of miR492 and corresponding downregulation of its target gene, CD147, which is implicated in VEGF production and the regulation of the mitochondrial apoptosis pathway [112]. Also significant seems to be the role of survivin inhibition caused by resveratrol, as confirmed by a study on SCC mice [113] and another work about melanoma cells and mice [114].

#### 2.2.5. Genistein and Skin Cancer

Genistein (GEN) is one of the most common isoflavones present in nature [115]. GEN exhibits various biological effects, such as chemopreventive and antitumor. It has been suggested in several studies that GEN has potential as a drug for skin cancer treatment, due to its different mechanisms of action and pronounced antioxidant activity [116,117]. Roh et al., with the aim of investigating the effects of orobol (a metabolite of GEN) on the prevention and treatment of chronic solar-simulated light (SSL)-induced SCC, developed mouse models that included both preventive and therapeutic approaches using SKH-1 hairless mice. The early administration of orobol was found to reduce the development of chronic, SSL-induced SCC. In addition, orobol was also found to have therapeutic efficacy in the early stages of tumors and was also efficient in reducing tumor regrowth after the treatment was discontinued. The authors demonstrated that orobol is capable of directly binding to T-LAK cell-originated protein kinase (TOPK), which is an oncogenic protein implicated in the development of SCC in an ATP-independent manner [118].

In some cancer cell lines, GEN can reduce the expression of cyclin D1, which is engaged in stop of the G2/M phase of the cell cycle [119]. Aprilliantina et al. investigated the role of GEN in the cyclin D1 expression in melanoma cells. The study found that low concentrations of GEN induced the upregulation of cyclin D1 expression in malignant melanoma cells, while high concentrations of GEN led to the downregulation of cyclin D1 expression. This biphasic action of genistein on cyclin D1 expression was found to be associated with the suppression of cell proliferation and subsequent apoptosis in malignant melanoma cells [119].

GEN has a low bioavailability when administered systemically [120]. Nanoemulsion (NE) formulations can be used to improve the dermal delivery and effectiveness of chemotherapeutic or preventive natural substances; in this sense, Brownlow et al. investigated the physicochemical characterization and photostability of an NE formulation using a surfactant mixture (Smix) of SolutolR HS-15 (SHS15) mixed with vitamin E TPGS (TPGS) as cosurfactant. They demonstrated that TocominR NE is a promising topical drug platform for skin photoprotection applications and that the use of NE formulations to improve the dermal release and efficacy of natural compounds such as GEN can enhance the solubility, bioavailability, and stability of poorly soluble natural substances, leading to enhanced therapeutic activity against skin conditions resulting from chronic exposure to harmful solar UVB radiation [121]. Permeation experiments conducted by Zampieri et al. showed that increased penetration was achieved when GEN-NCs were incorporated into a semi-solid gel formulation, indicating that GEN-NCs might be a promising nanocarrier system for the skin delivery of GEN [122].

### 2.3. Limits of the Study

According to the review of the literature, a potential use of these compounds for preventive/therapeutic purposes appears promising. However, the possible limitations of the study are:

- The precise mechanism by which these substances act has not been established and several pathways have been proposed that seem to confirm this property.
- Due to the variability in the studies performed, succeeding in homogenizing the results is not simple.
- The wide variability in dosages, delivery systems, and combinations of compounds used in the different studies may create bias.

For these reasons, further studies are needed to more accurately investigate the molecular pathways implicated and the target action of these nutraceuticals. Their potential therapeutic role in the primary prevention and complementary treatment of skin cancers makes this topic worthy of more attention.

In consideration of the increase in this type of tumors, it is essential to investigate new approaches to treating patients with the forms that are resistant to traditional therapy, or even reducing their incidence.

## 3. Conclusions

**Figure 1:**
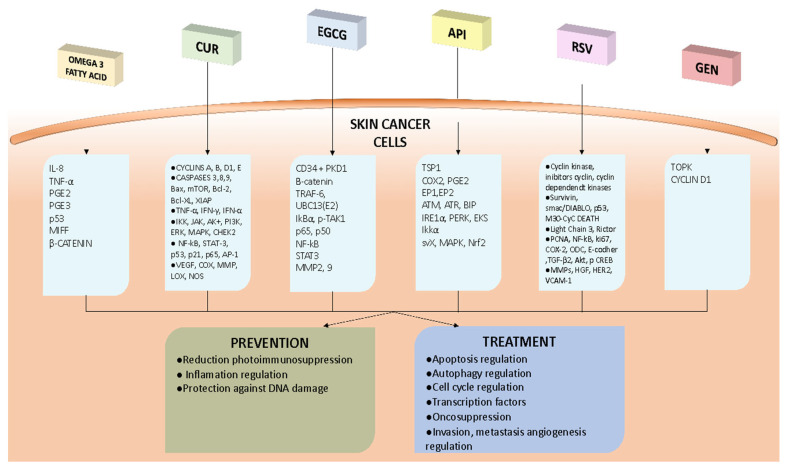
The figure shows a schematic representation of the cancer pathways that are activated by Omega 3 fatty acid, CUR, EGCG, API, RSV, and GEN. The diagram illustrates how these compounds stimulate pathways that can lead to prevention and treatment mechanism activated by these compounds.

Skin cancer is one of the most common types of malignancy and its incidence continues to rise globally. Although early detection and prevention are important strategies for reducing skin cancer risk, alternative approaches, such as chemoprevention and therapeutic interventions using natural compounds, have gained increasing attention in recent years. This point-to-point conclusion summarizes the potential chemopreventive and therapeutic effects of omega-3 fatty acids, curcumin, epigallocatechin gallate, apigenin, resveratrol, and genistein on skin cancer and their mechanisms of action (Figure 1), paying particular attention to the use of delivery systems to improve the bioavailability and efficacy of these compounds:

- Omega-3 fatty acids: these have been demonstrated to possess anti-tumor properties through the modulation of TNF-α, leading to a reduction in IL-8 expression and promoting a regulatory environment in the skin by reducing pro-inflammatory eicosanoids and increasing anti-inflammatory ones; oral ω-3 PUFAs appear to repeal photoimmunosuppression in human skin, giving support to their chemopreventive action; DHA inhibits melanoma cell growth, migration, and invasion, respectively, by increasing nuclear beta-catenin content and expression; to reduce exposure to environmental contaminants, it is recommended to consume fish in moderation and choose lower-mercury varieties (such as salmon, sardines, and trout).
- Curcumin: this plays an antitumor role in the skin by inhibiting cancer cell growth (inhibiting STAT3 and the G2/M checkpoint block,) and promoting apoptosis (modification of p53, reduction in Bcl-2, and increases in Bax, caspase-3, and -9) in both melanoma and NMSC; curcumin also appears to have a chemopreventive role in skin cancers and seems to have an adjuvant action in the photothermal therapy and sonodynamic therapy of melanoma by increasing ROS production; the challenge of the low bioavailability of curcumin can be overcome using liposomes, nanoparticles, and nanopattern films or by the use of analogs (e.g., DMC and DM-1); a combination of curcumin and tocopherol or siRNA seems to potentiate its antitumor action.
- Epigallocatechin gallate: this has shown potential in inhibiting skin tumors, inactivating β-catenin, and suppressing the TRAF6 E3 ubiquitin ligase activity in melanoma cells; the association of EGCG and metformin has a synergistic effect on melanoma cells; EGCG, encapsulated in different vesicular systems, also inhibits epidermoid carcinoma cell lines and reduces tumor sizes in mice.
- Apigenin: this could have a chemopreventive function, target TSP1, reduce the synthesis of COX-2, PGE2, EP1, and EP2, and restore the inhibition of autophagy in UVB-exposed human keratinocytes; apigenin could have a role in NMSC treatment, since it can regulate the mTOR pathway, inhibit IKKα, downregulate Srx, and reverse the hypermethylation of 15 CpG sites in the Nrf2 promoter; nanoparticles as carriers for apigenin represent a promising treatment for skin cancers, in particular NMSC.
- Resveratrol: this reduces cyclin D1 and increases cyclins A2, E1, Cdk1, and Cdk2, blocking tumor growth in melanoma; an important role is the promotion of apoptosis by resveratrol through the downregulation of Bcl-2 and FLIP; an inhibition of survivin by resveratrol appears to be implicated in the promotion of the apoptosis of both melanoma and NMSC cells; resveratrol could be a radiation sensitizer against melanoma; the use of nanostructured lipid carrier gels, solid lipid nanoparticles, and liposomes improves the release and bioavailability of resveratrol.
- Genistein: orobol (a metabolite of GEN) has been found to reduce the development of chronic, solar-simulated, light-induced skin cancer and to have therapeutic efficacy in the early stages of tumors by binding to TOPK, an oncogenic protein; a biphasic action of GEN on cyclin D1 expression was found to be associated with the suppression of cell proliferation and subsequent apoptosis in malignant melanoma cells; GEN has a low bioavailability when administered systemically, but NE formulations can improve the dermal delivery and efficacy of natural compounds such as GEN for skin photoprotection applications.
- Despite the limitations of the currently available data, the therapeutic role of these nutraceuticals seems clear. Further studies are needed to better understand how they can be exploited as an additional therapeutic tool.

## Data Availability

The data used to support the findings of this study can be made available by the corresponding author upon request.
